# Supplementary material for: Experience shapes the transformation of olfactory representations along the cortico-hippocampal pathway
Source: eLife. 2026 Jun 9;13:RP103373. doi: 10.7554/eLife.103373 (PMC13249424; doi:10.7554/eLife.103373)
Supplement: Peer review history

# Editor report: eLife Assessment

**DOI:** 10.7554/eLife.103373.3.sa0

**Authors:** Upinder Singh Bhalla

**Affiliations:** National Centre for Biological Sciences India

**Keywords:** Important, Solid

This **important** study describes the progressive transformation of olfactory information across five different brain regions in the olfactory pathway, including a comparison of responses to familiar and unfamiliar odors. This dataset is of broad interest for olfactory researchers and provides a **solid** analysis of a graded change in representations of odor identity and experience in different locations in the pathway.

# Referee report: Reviewer #1 (Public review)

**DOI:** 10.7554/eLife.103373.3.sa1

In this important study, the authors characterized the transformation of neural representations of olfactory stimuli from primary sensory cortex to multisensory regions in the medial temporal lobe and investigated how they were affected by non-associative learning. The authors used high-density silicon probe recordings from five different cortical regions while familiar vs. novel odors were presented to a head-restrained mouse. This is a timely study because unlike other sensory systems (e.g., vision), the progressive transformation of olfactory information is still poorly understood. The authors report that both odor identity and experience are encoded by all of these five cortical areas but nonetheless, some themes emerge. Single neuron tuning of odor identity is broad in the sensory cortices but becomes narrowly tuned in hippocampal regions. Furthermore, while experience affects neuronal response magnitudes in early sensory cortices, it changes the proportion of active neurons in hippocampal regions. Thus, this study is an important step forward in the ongoing quest to understand how olfactory information is progressively transformed along the olfactory pathway.

The study is well-executed. The direct comparison of neuronal representations from five different brain regions is impressive. Conclusions are based on single neuronal level as well as population level decoding analyses. Among all the reported results, one stands out for being remarkably robust. The authors show that the anterior olfactory nucleus (AON), which receives direct input from the olfactory bulb output neurons, was far superior at decoding odor identity as well as novelty compared to all the other brain regions. This is perhaps surprising because the other primary sensory region - the piriform cortex - has been thought to be the canonical site for representing odor identity. A vast majority of studies have focused on aPCx, but direct comparisons between odor coding in the AON and aPCx are rare. The experimental design of this current study allowed the authors to do so and the AON was found to convincingly outperform aPCx. Although this result goes against the canonical model, it is consistent with a few recent studies including one that predicted this outcome based on anatomical and functional comparisons between the AON-projecting tufted cells vs. the aPCx-projecting mitral cells in the olfactory bulb.

Future experiments are needed to probe the circuit mechanisms underlying the differential importance of the two primary olfactory cortices, as well as their potential causal roles in odor identification. Moreover, future work should test whether the decoding accuracy of odor identity and experience from neural data (as reported here) can predict the causal contributions of these regions, as revealed through perturbations during behavioral tasks that explicitly probe odor identification and/or experience.

# Referee report: Reviewer #2 (Public review)

**DOI:** 10.7554/eLife.103373.3.sa2

Summary:

This manuscript investigates how olfactory representations are transformed along the cortico-hippocampal pathway in mice during a non-associative learning paradigm involving novel and familiar odors. By recording single-unit activity in several key brain regions (AON, aPCx, LEC, CA1, and SUB), the authors aim to elucidate how stimulus identity and experience are encoded and how these representations change across the pathway.

The study addresses an important question in sensory neuroscience regarding the interplay between sensory processing and signaling novelty/familiarity. It provides insights into how the brain processes and retains sensory experiences, suggesting that the earlier stations in the olfactory pathway, the AON aPCx, play a central role in detecting novelty and encoding odor, while areas deeper into the pathway (LEC, CA1 & Sub) are more sparse and encodes odor identity but not novelty/familiarity. However, there are several concerns related to methodology, data interpretation, and the strength of the conclusions drawn.

Strengths:

The authors combine the use of modern tools to obtain high-density recordings from large populations of neurons at different stages of the olfactory system (although mostly one region at a time) with elegant data analyses to study an important and interesting question.

Weaknesses:

The first and biggest problem I have with this paper is that it is very confusing, and the results seem to be all over the place. In some parts, it seems like the AON and aPCx are more sensitive to novelty; in others, it seems the other way around. I find their metrics confusing and unconvincing. For example, the example cells in Figure 1C shows an AON neuron with a very low spontaneous firing rate and a CA1 with a much higher firing rate, but the opposite is true in Fig. 2A. So, what are we to make of Fig. 2C that shows the difference in firing rates between novel vs. familiar odors measured as a difference in spikes/sec. The meaning of this is unclear. The authors could have used a difference in Z-scored responses to normalize different baseline activity levels. (This is just one example of a problem with the methodology.)

There are a lot of high-level data analyses (e.g., decoding, analyzing decoding errors, calculating mutual information, calculating distances in state space, etc.) but very little neural data (except for Fig. 2C, and see my comment above about how this is flawed). So, if responses to novel vs. familiar odors are different in the AON and aPCx, how are they different? Why is decoding accuracy better for novel odors in CA1 but better for familiar odors in SUB (Fig. 3A)? The authors identify a small subset of neurons that have unusually high weights in the SVM analyses that contribute to decoding novelty, but they don't tell us which neurons these are and how they are responding differently to novel vs. familiar odors.

The authors call AON and aPCx "primary sensory cortices" and LEC, CA1, and Sub "multisensory areas". This is a straw man argument. For example, we now know that PCx encodes multimodal signals (Poo et al. 2021, Federman et al., 2024; Kehl et al., 2024), and LEC receives direct OB inputs, which has traditionally been the criterion for being considered a "primary olfactory cortical area". So, this terminology is outdated and wrong, and although it suits the authors' needs here in drawing distinctions, it is simplistic and not helpful moving forward.

Why not simply report z-scored firing rates for all neurons as a function of trial number? (e.g., Jacobson & Friedrich, 2018). Fig. 2C is not sufficient. For example, in the Discussion, they say, "novel stimuli caused larger increases in firing rates than familiar stimuli" (L. 270), but what does this mean? Odors typically increase the firing in some neurons and suppress firing in others. Where does the delta come from? Is this because novel odors more strongly activate neurons that increase their firing or because familiar odors more strongly suppress neurons?

Ls. 122-124 - If cells in AON and aPCx responded the same way to novel and familiar odors, then we would say that they only encode for odor and not at all for experience. So, I don't understand why the authors say these areas code for a "mixed representation of chemical identity and experience." "On the other hand," if LEC, CA1, and SUB are odor selective and only encode novel odors, then these areas, not AON and aPCx, are the jointly encoding chemical identity and experience. Also, I do not understand why, here, they say that AON and PCx respond to both while LEC, CA1, and SUB were selective for novel stimuli, but the authors then go on to argue that novelty is encoded in the AON and PCx, but not in the LEC, CA1, and SUB.

Ls. 132-140 - As presented in the text and the figure, this section is unclear and confusing. Their use of the word "shuffled" is a major source of this confusion, because this typically is the control that produces outcomes at chance level. More importantly, it seems as though they did the wrong analysis here. A better way to do this analysis is to train on some of the odors and test on an untrained odor (i.e., what Bernardi et al., 2021 called "cross-condition generalization performance"; CCGP).

Comments on revisions:

I think the authors have done an adequate job addressing the reviewers' concerns. Most importantly, I found the first version of the manuscript quite confusing, and the consequent clarifications have addressed this issue.

In several cases, I see their point, while I still disagree with whether they made the best decisions. However, the issues here do not fundamentally change the big-picture outcome, and if they want to dig in with their approaches (e.g., only using auROC or just reporting delta firing rates without any normalization), it's their choice.

# Referee report: Reviewer #3 (Public review)

**DOI:** 10.7554/eLife.103373.3.sa3

In this manuscript, the authors investigate how odor-evoked neural activity is modulated by experience within the olfactory-hippocampal network. The authors perform extracellular recordings in the anterior olfactory nucleus (AON), the anterior piriform (aPCx) and lateral entorhinal cortex (LEC), the hippocampus (CA1) and the subiculum (SUB), in naïve mice and in mice repeatedly exposed to the same odorants. They determine the response properties of individual neurons and use population decoding analyses to assess the effect of experience on odor information coding across these regions.

The authors' findings show that odor identity is represented in all recorded areas, but that the response magnitude and selectivity of neurons are differentially modulated by experience across the olfactory-hippocampal pathway.

Overall, this work represents a valuable multi-region data set of odor-evoked neural activity. However, a few limitations in experimental design and analysis restrict the conclusions that can be drawn from this study.

Main limitations:

The authors use a non-associative learning paradigm - repeated odor exposure - to test how experience modulates odor responses along the olfactory-hippocampal pathway. While repeated odor exposure clearly modulates sampling behavior and odor-evoked neural activity, the relevance of this modulation across different brain areas remains difficult to assess.

The authors discuss the olfactory-hippocampal pathway as a transition from primary sensory (AON, aPCx) to associative areas (LEC, CA1, SUB). While this is reasonable, given the known circuit connectivity, other interpretations are possible. For example, AON, aPCx, and LEC receive direct inputs from the olfactory bulb ('primary cortex'), while CA1 and SUB do not; AON receives direct top-down inputs from CA1 ('associative cortex'), while aPCx does not. In fact, the data presented in this manuscript do not appear to support a consistent transformation from sensory to associative, as implied by the authors.

# Author comment: Author response

**DOI:** 10.7554/eLife.103373.3.sa4

**Authors:** Schiltz Eleonore, Broux Martijn, Aydin Cagatay, Pedro J Gonçalves, Sebastian Haesler

**Affiliations:** Neuroelectronics Research Flanders Leuven Belgium; Neuroelectronics Research Flanders Leuven Belgium; Neuroelectronics Research Flanders Leuven Belgium; Neuro-Electronics Research Flanders Leuven Belgium; Neuroelectronics Research Flanders Leuven Belgium

The following is the authors’ response to the original reviews.

**Public reviews:**

**Reviewer #1 (Public review):**

In this important study, the authors characterized the transformation of neural representations of olfactory stimuli from the primary sensory cortex to multisensory regions in the medial temporal lobe and investigated how they were affected by non-associative learning. The authors used high-density silicon probe recordings from five different cortical regions while familiar vs. novel odors were presented to a head-restrained mouse. This is a timely study because unlike other sensory systems (e.g., vision), the progressive transformation of olfactory information is still poorly understood. The authors report that both odor identity and experience are encoded by all of these five cortical areas but nonetheless some themes emerge. Single neuron tuning of odor identity is broad in the sensory cortices but becomes narrowly tuned in hippocampal regions. Furthermore, while experience affects neuronal response magnitudes in early sensory cortices, it changes the proportion of active neurons in hippocampal regions. Thus, this study is an important step forward in the ongoing quest to understand how olfactory information is progressively transformed along the olfactory pathway.

The study is well-executed. The direct comparison of neuronal representations from five different brain regions is impressive. Conclusions are based on single neuronal level as well as population level decoding analyses. Among all the reported results, one stands out for being remarkably robust. The authors show that the anterior olfactory nucleus (AON), which receives direct input from the olfactory bulb output neurons, was far superior at decoding odor identity as well as novelty compared to all the other brain regions. This is perhaps surprising because the other primary sensory region - the piriform cortex - has been thought to be the canonical site for representing odor identity. A vast majority of studies have focused on aPCx, but direct comparisons between odor coding in the AON and aPCx are rare. The experimental design of this current study allowed the authors to do so and the AON was found to convincingly outperform aPCx. Although this result goes against the canonical model, it is consistent with a few recent studies including one that predicted this outcome based on anatomical and functional comparisons between the AON-projecting tufted cells vs. the aPCx-projecting mitral cells in the olfactory bulb (Chae, Banerjee et. al. 2022). Future experiments are needed to probe the circuit mechanisms that generate this important difference between the two primary olfactory cortices as well as their potential causal roles in odor identification.

The authors were also interested in how familiarity vs. novelty affects neuronal representation across all these brain regions. One weakness of this study is that neuronal responses were not measured during the process of habituation. Neuronal responses were measured after four days of daily exposure to a few odors (familiar) and then some other novel odors were introduced. This creates a confound because the novel vs. familiar stimuli are different odorants and that itself can lead to drastic differences in evoked neural responses. Although the authors try to rule out this confound by doing a clever decoding and Euclidian distance analysis, an alternate more straightforward strategy would have been to measure neuronal activity for each odorant during the process of habituation.

**Reviewer #2 (Public review):**

This manuscript investigates how olfactory representations are transformed along the cortico-hippocampal pathway in mice during a non-associative learning paradigm involving novel and familiar odors. By recording single-unit activity in several key brain regions (AON, aPCx, LEC, CA1, and SUB), the authors aim to elucidate how stimulus identity and experience are encoded and how these representations change across the pathway.

The study addresses an important question in sensory neuroscience regarding the interplay between sensory processing and signaling novelty/familiarity. It provides insights into how the brain processes and retains sensory experiences, suggesting that the earlier stations in the olfactory pathway, the AON aPCx, play a central role in detecting novelty and encoding odor, while areas deeper into the pathway (LEC, CA1 & Sub) are more sparse and encodes odor identity but not novelty/familiarity. However, there are several concerns related to methodology, data interpretation, and the strength of the conclusions drawn.

Strengths:

The authors combine the use of modern tools to obtain high-density recordings from large populations of neurons at different stages of the olfactory system (although mostly one region at a time) with elegant data analyses to study an important and interesting question.

Weaknesses:

(1) The first and biggest problem I have with this paper is that it is very confusing, and the results seem to be all over the place. In some parts, it seems like the AON and aPCx are more sensitive to novelty; in others, it seems the other way around. I find their metrics confusing and unconvincing. For example, the example cells in Figure 1C show an AON neuron with a very low spontaneous firing rate and a CA1 with a much higher firing rate, but the opposite is true in Figure 2A. So, what are we to make of Figure 2C that shows the difference in firing rates between novel vs. familiar odors measured as a difference in spikes/sec. This seems nearly meaningless. The authors could have used a difference in Z-scored responses to normalize different baseline activity levels. (This is just one example of a problem with the methodology.)

We appreciate the reviewer’s concerns regarding clarity and methodology. It is less clear why all neurons in a given brain area should have similar firing rates. Anatomically defined brain areas typically comprise of multiple cell types, which can have diverse baseline firing rates. Since we computed absolute firing rate differences per neuron (i.e., novel vs. familiar odor responses within the same neuron), baseline differences across neurons do not have a major impact.

The suggestion to use Z-scores instead of absolute firing rate differences is well taken. However, Z-scoring assumes that the underlying data are normally distributed, which is not the case in our dataset. Specifically, when analyzing odor-evoked firing rates on a per-neuron basis, only 4% of neurons exhibit a normal distribution. In cases of skewed distributions, Z-scoring can distort the data by exaggerating small variations, leading to misleading conclusions. We acknowledge that different analysis methods exist, we believe that our chosen approach best reflects the properties of the dataset and avoids potential misinterpretations introduced by inappropriate normalization techniques.

(2) There are a lot of high-level data analyses (e.g., decoding, analyzing decoding errors, calculating mutual information, calculating distances in state space, etc.) but very little neural data (except for Figure 2C, and see my comment above about how this is flawed). So, if responses to novel vs. familiar odors are different in the AON and aPCx, how are they different? Why is decoding accuracy better for novel odors in CA1 but better for familiar odors in SUB (Figure 3A)? The authors identify a small subset of neurons that have unusually high weights in the SVM analyses that contribute to decoding novelty, but they don't tell us which neurons these are and how they are responding differently to novel vs. familiar odors.

We performed additional analyses to address the reviewer’s feedback (Figures 2C-E and lines 118-132) and added more single-neuron data (Figures 1, S3 and S4).

(3) The authors call AON and aPCx "primary sensory cortices" and LEC, CA1, and Sub "multisensory areas". This is a straw man argument. For example, we now know that PCx encodes multimodal signals (Poo et al. 2021, Federman et al., 2024; Kehl et al., 2024), and LEC receives direct OB inputs, which has traditionally been the criterion for being considered a "primary olfactory cortical area". So, this terminology is outdated and wrong, and although it suits the authors' needs here in drawing distinctions, it is simplistic and not helpful moving forward.

We appreciate the reviewer’s concern regarding the classification of brain regions as “primary sensory” versus “multisensory.” Of note, the cited studies (Poo et al., 2021; Federman et al., 2024; Kehl et al., 2024) focus on posterior PCx (pPCx), while our recordings were conducted in very anterior section of anterior PCx. The aPCx and pPCx have distinct patterns of connectivity, both anatomically and functionally. To the best of our knowledge, there is no evidence for multimodal responses in aPCx, whereas there is for LEC, CA1 and SUB. Furthermore, our distinction is not based on a connectivity argument, as the reviewer suggests, but on differences in the α-Poisson ratio (Figure 1E and F).

To avoid confusion due to definitions of what constitutes a “primary sensory” region, we adopted a more neutral description throughout the manuscript.

(4) Why not simply report z-scored firing rates for all neurons as a function of trial number? (e.g., Jacobson & Friedrich, 2018). Figure 2C is not sufficient.

Regarding z-scores, please see response to 1). We further added a figure showing responses of all neurons to novel stimuli (using ROC instead of z-scoring, as described previously (e.g. Cohen et al. Nature 2012). We added the following figure to the supplementary for the completeness of the analysis (S2E).

For example, in the Discussion, they say, "novel stimuli caused larger increases in firing rates than familiar stimuli" (L. 270), but what does this mean?

This means that on average, the population of neurons exhibit higher firing rates in response to novel odors compared to familiar ones.

Odors typically increase the firing in some neurons and suppress firing in others. Where does the delta come from? Is this because novel odors more strongly activate neurons that increase their firing or because familiar odors more strongly suppress neurons?

We thank the reviewer for this valuable feedback and extended the characterization of firing rate properties, including a separate analysis of neurons (i) significantly excited by odorants, (ii) significantly inhibited by odorants and (iii) not responsive to odorants. We added the analysis and corresponding discussion to the main manuscript (Figures 2C-E and lines 118-132)

(5) Lines 122-124 - If cells in AON and aPCx responded the same way to novel and familiar odors, then we would say that they only encode for odor and not at all for experience. So, I don't understand why the authors say these areas code for a "mixed representation of chemical identity and experience." "On the other hand," if LEC, CA1, and SUB are odor selective and only encode novel odors, then these areas, not AON and aPCx, are the jointly encoding chemical identity and experience. Also, I do not understand why, here, they say that AON and PCx respond to both while LEC, CA1, and SUB were selective for novel stimuli, but the authors then go on to argue that novelty is encoded in the AON and PCx, but not in the LEC, CA1, and SUB.

We appreciate the reviewer’s request for clarification. Throughout the brain areas we studied, odorant identity and experience can be decoded. However, the way information is represented is different between regions. We acknowledge that that “mixed” representation is a misleading term and removed it from the manuscript.

In AON and aPCx, neurons significantly respond to both novel and familiar odors. However, the magnitude of their responses to novel and familiar odors is sufficiently distinct to allow for decoding of odor experience (i.e., whether an odor is novel or familiar). Moreover, novelty engages more neurons in encoding the stimulus (Figure 2D). In neural space, the position of an odor’s representation in AON and aPCx shifts depending on whether it is novel or familiar, meaning that experience modifies the neural representation of odor identity. This suggests that in these regions the two representations are intertwined.

In contrast, some neurons in LEC, CA1, and SUB exhibit responses to novel odors, but few neurons respond to familiar odors at all. This suggests a more selective encoding of novelty.

(6) Lines 132-140 - As presented in the text and the figure, this section is poorly written and confusing. Their use of the word "shuffled" is a major source of this confusion, because this typically is the control that produces outcomes at the chance level. More importantly, they did the wrong analysis here. The better and, I think, the only way to do this analysis correctly is to train on some of the odors and test on an untrained odor (i.e., what Bernardi et al., 2021 called "cross-condition generalization performance"; CCGP).

We appreciate the feedback and thank the reviewer for the recommendation to implement cross-condition generalization performance (CCGP) as used in Bernardi et al., 2020. We acknowledge that the term "shuffled" may have caused confusion, as it typically refers to control analyses producing chance-level outcomes. In our case, by "shuffling" we shuffled the identity of novel and familiar odors to assess how much the decoder relies on odor identity when distinguishing novelty. This test provided insight into how novelty-based structure exists within neural activity beyond random grouping but does not directly assess generalization.

As suggested, we used CCGP to measure how well novelty-related representations generalize across different odors. Our findings show that in AON and aPCx, novelty-related information is indeed highly generalizable, supporting the idea that these regions encode novelty in a less odor-selective manner (Figure 2K).

**Reviewer #3 (Public review):**

In this manuscript, the authors investigate how odor-evoked neural activity is modulated by experience within the olfactory-hippocampal network. The authors perform extracellular recordings in the anterior olfactory nucleus (AON), the anterior piriform (aPCx) and lateral entorhinal cortex (LEC), the hippocampus (CA1), and the subiculum (SUB), in naïve mice and in mice repeatedly exposed to the same odorants. They determine the response properties of individual neurons and use population decoding analyses to assess the effect of experience on odor information coding across these regions.

The authors' findings show that odor identity is represented in all recorded areas, but that the response magnitude and selectivity of neurons are differentially modulated by experience across the olfactory-hippocampal pathway.

Overall, this work represents a valuable multi-region data set of odor-evoked neural activity. However, limitations in the interpretability of odor experience of the behavioral paradigm, and limitations in experimental design and analysis, restrict the conclusions that can be drawn from this study.

**Recommendations for the authors:**

**Reviewer #1 (Recommendations for the authors):**

Some suggestions, in no particular order, to further improve the manuscript:

(1) The example neuronal responses for CA1 and SUB in Figure 1 are not very inspiring. To my eyes, the odor period response is not that different from the baseline period. In general, a thorough characterization of firing rate properties during the odor period between the different brain regions would be informative.

We thank the reviewer for this valuable feedback. We have replaced the example neurons from CA1 and SUB in Figure 1C. We further extended the characterization of firing rate properties, including a separate analysis of neurons (i) significantly excited by odorants, (ii) significantly inhibited by odorants and (iii) not responsive to odorants. We added the analysis and corresponding discussion to the main manuscript (Figures 2C-E and lines 118-132)

(2) For the summary in Figure 1, why not show neuronal responses as z-scored firing rates as opposed to auROC?

We chose to use auROC instead of z-scored firing rates due to the non-normality of the dataset, which can distort results when using z-scores. Specifically, z-scoring can exaggerate small deviations in neurons with low responsiveness, potentially leading to misleading conclusions. auROC provides a more robust measure of response change that is less sensitive to these distortions because it does not assume any specific distribution. This approach has been used previously (e.g. Cohen et al. 2012, Nature).

(3) To study novelty, the authors presented odorants that were not used during four days of habituation. But this design makes it hard to dissociate odor identity from novelty. Why not track the response of the same odorants during the habituation process itself?

We respectfully disagree with the argument that using different stimuli as novel and familiar constitutes a confound in our analysis. In our study, we used multiple different, structurally dissimilar single molecule chemicals which were randomly assigned to novel and familiar categories in each animal. If individual stimuli did cause “drastic differences in evoked neural responses”, these would be evenly distributed between novel and familiar stimuli. It is therefore extremely unlikely that the clear differences we observed between novel and familiar conditions and between brain areas can be attributed to the contribution of individual stimuli, in particular given our analyses was performed at the population level. In fact, we observed that responses between novel and familiar conditions were qualitatively very similar in the short time window after odor onset (Figure 1G and H).

Importantly, the goal of this study was to investigate the impact of long-term habituation over more than 4 days, rather than short term habituation during one behavioral session. However, tracking the activity of large numbers of neurons across multiple days presents a significant technical challenge, due to the difficulty of identifying stable single-unit recordings over extended periods of time with sufficient certainty. Tools that facilitate tracking have recently been developed (e.g. Yuan AX et al., Elife. 2024) and it will be interesting to apply them to our dataset in the future.

(4) Since novel odors lead to greater sniffing and sniffing strongly influences firing rates in the olfactory system, the authors decided to focus on a 400 ms window with similar sniffing rates for both novel vs. familiar odors. Although I understand the rationale for this choice, I worry that this is too restrictive, and it may not capture the full extent of the phenomenology.

Could the authors model the effect of sniffing on firing rates of individual neurons from the data, and then check whether the odor response for novel context can be fully explained just by increased sniffing or not?

It is an interesting suggestion to extend the window of analysis and observe how responses evolve with sniffing (and other behavioral reactions). To address this, we added an additional figure to the supplementary material, showing the mean responses of all neurons to novel stimuli during the entire odor presentation window (Fig. S1B).

As suggested, we further created a Generalized Linear Model (GLM) for the entire 2s odor stimulation period, incorporating sniffing and novelty as independent variables. As expected, sniffing had a dominant impact on firing rate in all brain areas. A smaller proportion of neurons was modulated by novelty or by the interaction between novelty x breathing, suggesting the entrainment of neural activity by sniffing during the response to novel odors. These results support our decision to focus the analysis on the early 400ms window in order to dissociate the effects of novelty and behavioral responses. Taken together, our results suggest that odorant responses are modulated by novelty early during odorant processing, whereas at later stages sniffing becomes the predominant factor driving firing (Figure S2C-D).

(5) The authors conclude that aPCx has a subset of neurons dedicated to familiar odors based on the distribution of SVM weights in Figure 3D. To me, this is the weakest conclusion of the paper because although significant, the effect size is paltry; the central tendencies are hardly different for the two conditions in aPCx. Could the authors show the PSTHs of some of these neurons to make this point more convincing?

We appreciate the reviewer’s concern regarding the effect size. To strengthen our conclusion, we now include PSTHs of representative neurons in the least 10% and best 10% of neuronal population based on the SVM analysis (Figures S3 and S4). We hope this provides more clarity and support for the interpretation that there is a subset of neurons in aPCx that show greater sensitivity to familiar odors, despite the relatively modest central tendency differences.

In the revised manuscript, we discuss the effect size more explicitly in the text to provide context for its significance (lines 193 - 195).

**Reviewer #2 (Recommendations for the authors):**

(1) The authors only talk about "responsive" neurons. Does this include neurons whose activity increases significantly (activated) and neurons whose activity decreases (suppressed)?

Yes, the term "responsive" refers to neurons whose activity either increases significantly (excited) or decreases (inhibited) in response to the odor stimuli. We performed additional analyses to characterize responses separately for the different groups (Figure 2C-E and lines 118-132).

(2) Line 54 - The Schoonover paper doesn't show that cells lose their responses to odors, but rather that the population of cells that respond to odors changes with time. That is, population responses don't become more sparse

The fact that “the population of cells that respond to odors changes with time”, implies that some neurons lose their responsiveness (e.g. unit 2 in Figure 1 of Schoonover et al., 2021), while others become responsive (e.g. unit 1 in Figure 1 of Schoonover et al., 2021). Frequent responses reduce drift rate (Figure 4 of Schoonover et al., 2021), thus fewer neurons loose or gain responsiveness. We have revised the manuscript to clarify this.

(3) Line 104 - "Recurrent" is incorrectly used here. I think the authors mean "repeated" or something more like that.

Thank you for pointing this out. We replaced "recurrent" with "repeated".

(4) Figure 3D - What is the scale bar here?

We apologize for the accidental omission. The scale bar was be added to Figure 3D in the revised version of the manuscript.

(5) Line 377 - They say they lowered their electrodes to "200 um/s per second." This must be incorrect. Is this just a typo, or is it really 200 um/s, because that's really fast?

Thank you for pointing this out. It was 20 to 60 um/s, the change has been made in the manuscript.

(6) Line 431: The authors say they used auROC to calculate changes in firing rates (which I think is only shown in Figure 1D). Note that auROC measures the discriminability of two distributions, not the strength or change in the strength of response.

Indeed we used auROC to measure the discriminability of firing between baseline and during stimulus response. We have corrected the wording in the methods.

(7) Figure 1B: The anatomical locations of the five areas they recorded from are straightforward, and this figure is not hugely helpful. However, the reader would benefit tremendously by including an experimental schematic. As is, we needed to scour the text and methods sections to understand exactly what they did when.

We thank the reviewer for this suggestion. We included an experimental schematic in the supplementary material.

(8) Figure 1F(left): This plot is much less useful without showing a pre-odor window, even if only times after the odor onset were used for calculation alpha

We appreciate this concern, however the goal of Figure 1F is to illustrate the meaning of the alpha value itself. We chose not to include a pre-odor window comparison to avoid confusing the reader.

(9) Figure 2A: What are the bar plots above the raster plots? Are these firing rates? Are the bars overlaid or stacked? Where is the y-axis scale bar?

The bar plots above the raster plots represent a histogram of the spike count/trials over time, with a bin width of 50 ms. These bars are overlaid on the raster plot. We will include a y-axis scale bar in the revised figure to clarify the presentation.

(10) Figure 4G: This makes no sense. First, the Y axis is supposed to measure standard deviation, but the axis label is spikes/s. Second, if responses in the AON are much less reliable than responses in "deeper" areas, why is odor decoding in AON so much better than in the other areas?

We acknowledge the error in the axis label, and we will correct it to indicate the correct units. AON has a larger response variability but also larger responses magnitudes, which can explain the higher decoding accuracy.

(11) From the model and text, one predicts that the lifetime sparseness increases along the pathway. The authors should use this metric as well/instead of "odor selectivity" because of problems with arbitrary thresholding.

We acknowledge that lifetime sparseness, often computed using lifetime kurtosis, can be an informative measure of selectivity. However, we believe it has limitations that make it less suitable for our analysis. One key issue is that lifetime sparseness does not account for the stability of responses across multiple presentations of the same stimulus. In contrast, our odor selectivity measure incorporates trial-to-trial variability by considering responses over 10 trials and assessing significance using a Wilcoxon test compared to baseline. While the choice of a p-value threshold (e.g., 0.05) is somewhat arbitrary, it is a widely accepted statistical convention. Additionally, lifetime sparseness does not account for excitatory and inhibitory responses. For example, if a neuron X is strongly inhibited by odor A, strongly excited by odor B, and unresponsive to odors C and D, lifetime sparseness would classify it as highly selective for odor B, without capturing its inhibitory selectivity for odor A. The lifetime sparseness will be higher than if X was simply unresponsive for A.

Our odor selectivity measure addresses this by considering both excitation and inhibition as potential responses. Thus, while lifetime sparseness could provide a useful complementary perspective in another type of dataset, it does not fully capture the dynamics of odor selectivity here.

**Author response 1.**

**Author response image 1.** Lifetime Kurtosis distribution per region.

**Reviewer #3 (Recommendations for the authors):**

Main points:

(1) The authors use a non-associative learning paradigm - repeated odor exposure - to test how experience modulates odor responses along the olfactory-hippocampal pathway. While repeated odor exposure clearly modulates odor-evoked neural activity, the relevance of this modulation and its differential effect across different brain areas are difficult to assess in the absence of any behavioral read-outs.

Our experimental paradigm involves a robust, reliable behavioral readout of non-associative learning. Novel olfactory stimuli evoke a well-characterized orienting reaction, which includes a multitude of physiological reactions, including exploratory sniffing, facial movements and pupil dilation (Modirshanechi et al., Trends Neuroscience 2023). In our study, we focused on exploration sniffing.

Compared to associative learning, non-associative learning might have received less attention. However, it is critically important because it forms the foundation for how organisms adapt to their environment through experience without forming associations. This is highlighted by the fact that non-instrumental stimuli can be remembered in large number (Standing, 1973) and with remarkable detail (Brady et al., 2008). While non-associative learning can thus create vast, implicit memory of stimuli in the environment, it is unclear how stimulus representations reflect this memory. Our study contributes to answering this question. We describe the impact of experience on olfactory sensory representations and reveal a transformation of representations from olfactory cortical to hippocampal structures. Our findings also indicate that sensory responses to familiar stimuli persist within sensory cortical and hippocampal regions, even after spontaneous orienting behaviors habituated. Further studies involving experimental manipulation techniques are needed to elucidate the causal mechanisms underlying the formation of stimulus memory during non-associative learning.

(2) The authors discuss the olfactory-hippocampal pathway as a transition from primary sensory (AON, aPCx) to associative areas (LEC, CA1, SUB). While this is reasonable, given the known circuit connectivity, other interpretations are possible. For example, AON, aPCx, and LEC receive direct inputs from the olfactory bulb ('primary cortex'), while CA1 and SUB do not; AON receives direct top-down inputs from CA1 ('associative cortex'), while aPCx does not. In fact, the data presented in this manuscript does not appear to support a consistent, smooth transformation from sensory to associative, as implied by the authors (e.g. Figure 4A, F, and G).

Thank you for this insightful comment. Indeed, there are complexities in the circuitry, and the relationships between different areas are not linear. We believe that AON and aPCx are distinctly different from LEC, CA1 and SUB, as the latter areas have been shown to integrate multimodal sensory information. To avoid confusion due to definitions of what constitutes a “primary sensory” region, we adopted a more neutral description throughout the manuscript. We also removed the term “gradual” to describe the transition of neural representations from olfactory cortical to hippocampal areas.

(3) The analysis of odor-evoked responses is focused on a 400 ms window to exclude differences in sniffing behavior. This window spans 200 ms before and after the first inhalation after odor onset. Inhalation onset initiates neural odor responses - why do the authors include neural data before inhalation onset?

The reason to include a brief time window prior to odor onset is to account for what is often called “partical” sniffs. In our experimental setup, odor delivery is not triggered by the animal’s inhalation. Therefore, it can happen that an animal has just begun to inhale when the stimulus is delivered. In this case, the animal is exposed to odorant molecules prior to the first complete inhalation after odor onset. We acknowledge that this limits the temporal resolution of our measurements, but it does not affect the comparison of sensory representations between different brain areas.

It would also be interesting to explore the effect of sniffing behavior (see point 2) on odor-evoked neural activity.

Thank you for your comment, we performed additional analysis including a GLM to address this question (Figure S2C-D).

Minor points:

(4) Figure 2A represents raster plots for 2 neurons per area - it is unclear how to distinguish between the 2 neurons in the plots.

Figure 2A shows one example neuron per brain area. Each neurons has two raster plot which indicate responses to either a novel (orange) or a familiar stimulus (blue). We have revised the figure caption for clarity.

(5) Overall, axes should be kept consistent and labeled in more detail. For example, Figure 2H and I are difficult to compare, given that the y-axis changes and that decoding accuracies are difficult to estimate without additional marks on the y-axis.

Axes are indeed different, because chance level decoding accuracy is different between those two figures. The decoding between novel and familiar odors has a chance level of 0.5, while chance level decoding odors is 0.1 (there are 10 odors to decode the identity from).

(6) Some parts of the discussion seem only loosely related to the data presented in this manuscript. For example, the statement that 'AON rather than aPCx should be considered as the primary sensory cortex in olfaction' seems out of context. Similarly, it would be helpful to provide data on the stability of subpopulations of neurons tuned to familiar odors, rather than simply speculate that they could be stable. The authors could summarize more speculative statements in an 'Ideas and Speculation' subsection.

Thank you for your comment. We appreciate your perspective on our hypotheses. We have revised the discussion accordingly. Specifically, we removed the discussion of stable subpopulations, since we have not performed longitudinal tracking in this study.

(7) The authors should try to reference relevant published work more comprehensively.

Thank you for your comment. We attempted to include relevant published work without exceeding the limit for references but might have overseen important contributions. We apologize to our colleagues, whose relevant work might not have been cited.
